# Monthly Summary

**Published:** 1885-12

**Authors:** 


### Monthly Summary.

---

Sore Mouth under Plates.—Dr. G. V. Black says: "The subject of sore mouths found under rubber plates should be further investigated. This condition has been attributed to the coloring matter of red rubber plates, but it appears under metal and porcelain plates also. A partial study of the causes of this disease has led to the discovery of leucocytes and micro-organisms under plates. In examining scrapings from plates and gums I have been surprised at the abundance of these organisms. If more abundant under rubber than under metal or porcelain plates, I am disposed to attribute the fact to the rougher surfaces of the rubber plates, which afforded greater facilities for the lodgment of foreign substances of a nature favorable to the growth of the organisms. Wandering cells—leucocytes—were seen constantly changing forms, micrococci aggregating around and among them. The wandering cells take up and digest the micrococci and thus aid in preventing the increase of the latter. Cleanliness is the chief preventive. The condition described differs from that of mercurial poisoning. I do not regard the coloring matter injurious. The temperature of the tissues under a plate of non-conducting material varies from the normal less than one degree. Plates are not cleansed frequently enough.—*Items of Interest.*

